# A Large Ovarian Tumor

**Published:** 1892-12

**Authors:** Mary A. Dixon Jones

**Affiliations:** 163 DeKalb Avenue; Brooklyn, N. Y.


### A LARGE OVARIAN TUMOR.

By MARY A. DIXON JONES, M. D., Brooklyn, N. Y.

In the September number of the Journal is an interesting account of the removal of an Ovarian Tumor of Unusual Size, by Charles H. Richmond, M. D. Allow me also to report the successful removal of an ovarian tumor of large size, though not weighing quite as many pounds as the one mentioned by Dr. Richmond, which "weighed fully eighty pounds." In my case the cyst contents weighed near sixty pounds.

The patient, Mrs. N., first consulted me in March, 1889 ; abdomen enormously enlarged ; said, "for the past six years her life had been a burden to herself and family." Some months previously she had consulted a physician, who told her she had dropsy, and treated her for the same. Subsequently, she consulted another physician, who "said she had a very large tumor, and the only possible relief was an operation."

The father of the patient advised her to see a woman for whom I had performed an operation a year previously. She did so, and this patient of mine strongly urged Mrs. N. to consult me. I diagnosticated at once an ovarian tumor, and told the patient it should be removed without delay. She was anxious to have this done, and entered the Woman's Hospital, of Brooklyn, March 27, 1889.

A few days after, everything was prepared for the operation, but at the last moment the patient's courage failed. She was so nervous that I told her she could put it off, or, indeed, not have the operation at all unless she wished. Before another week had passed, she was more anxious than ever that it should be performed.

There were the usual preparations as for abdominal section—perfect antisepsis in everything. I first made an incision of about three inches in the tense abdominal walls. The sac was exposed ; I introduced a trocar, drew off the fluid, removed the cyst, flushed well the peritoneal cavity, and closed the abdominal wound.

The whole operation was concluded in less than half an hour. The patient made an excellent and rapid recovery. The wound healed readily, and the patient was able to leave the hospital on the 18th of April, as she said, "a well and strong woman."

163 DeKalb Avenue.

